# Optimizing IELTS Writing Task 2 performance: effects of the WeCWI-Enabled Tencent Applications (WETA) intervention, UTAUT-validated user acceptance, and implied motivational-affective benefits

**DOI:** 10.3389/fpsyg.2026.1864021

**Published:** 2026-07-20

**Authors:** Changbao Suo, Jiashun Yu, Boyi Li, Boon Yih Mah

**Affiliations:** 1 Academy of Language Studies, Universiti Teknologi MARA, Cawangan Pulau Pinang, Permatang Pauh, Pulau Pinang, Malaysia; 2 School of Foreign Studies, Hebei University of Architecture, Zhangjiakou, Hebei, China

**Keywords:** educational equity, EFL learning and instruction, IELTS writing, motivational-affective benefits, Technology-Enhanced Language Learning (TELL), UTAUT model, WeCWI, WETA

## Abstract

**Introduction:**

Though Technology Enhanced Language Learning (TELL) has advanced EFL writing instruction, existing work remains Western-centric, lacking validated, context-appropriate tools for Chinese learners’ persistent IELTS Writing Task 2 challenges. Furthermore, studies have separated writing performance from user acceptance, while the motivational and affective impacts of such interventions remain underexplored in Asia-Pacific EFL settings. To meet the challenges, this study designed, implemented and assessed WeCWI-Enabled Tencent Applications (WETA)—a blended TELL tool designed to integrate WeChat, Tencent Meeting and Web-based Cognitive Writing Instruction (WeCWI), and analyzed its influence on user acceptance based on the IELTS Writing Task 2 performance and Unified Theory of Acceptance and Use of Technology (UTAUT) model.

**Methods:**

Using an explanatory sequential mixed-methods approach, 60 Chinese EFL undergraduates were divided for an 8-week quasi-experiment.

**Results:**

The ANCOVA results showed that the intervention group’s participants were associated with a significant and large effect gain in writing scores and also showed gains in its four IELTS scoring sub-criteria, especially in Lexical Resources. Thematic analysis of semi-structured interviews confirmed high user acceptance and strong sustained use intention, driven by all core UTAUT constructs.

**Discussion:**

Qualitative reflections from participants implied that WETA’s design suggests a potential for mitigating high-stakes writing anxiety and is associated with increased academic self-efficacy, providing hypothesis-generating motivational-affective insights for the Asia-Pacific EFL context. These findings demonstrate the feasibility of the proposed WeCWI-ADDIE-UTAUT integrated framework, and suggest WETA as a low-cost, context-appropriate, and equitable TELL solution outperforming conventional instruction for Chinese EFL undergraduates.

## 1 Introduction

Against the backdrop of global digital transformation, Technology-Enhanced Language Learning (TELL) has emerged as a transformative force in EFL writing instruction. Extensive research documents its benefits for learner-centered practice, collaborative learning, and personalized skill development (Fathi et al., 2019; Fu and Wang, 2020; Sun and Asmawi, 2023; Yan, 2019). Concurrently, educational psychology establishes emotion and motivation as core determinants of sustained language learning outcomes. In exam-driven Asia-Pacific contexts, high-stakes writing anxiety and low self-efficacy remain pervasive yet under-addressed barriers (Bandura, 1997). For Chinese IELTS test-takers, this dual challenge is particularly acute. Global data consistently show they underperform in Writing Task 2, with a 2022 mean score of just 5.8. Both linguistic deficits and unaddressed affective barriers limit their sustained growth (IELTS, n.d.; Liu and Deng, 2019; Suo et al., 2025b). Traditional teacher-centered instruction fails to resolve persistent weaknesses across the four IELTS scoring criteria: Task Response (TR), Coherence and Cohesion (CC), Lexical Resources (LR), and Grammatical Range and Accuracy (GRA) (IELTS, n.d.). It also overlooks the critical links between learning design, affective experiences, and long-term performance gains (Liu and Deng, 2019; Yan, 2019). These persistent pedagogical gaps leave learners struggling with inadequate prompt analysis, underdeveloped arguments, illogical text structure, repetitive vocabulary, and persistent grammatical errors, while exacerbating negative affective states (Chen, 2019; Liang, 2024; Moore et al., 2016; Suo et al., 2025b).

While TELL offers a promising pathway to address these gaps, existing scholarship has three critical, interconnected limitations. First, most TELL research centers on Western commercial platforms. Scant attention is paid to locally available tools aligned with Chinese learners’ daily digital ecosystem. This ecosystem is overwhelmingly dominated by Tencent’s WeChat and Tencent Meeting, used by 90% of university teachers and students (Qin and Yu, 2024; Wang and Luo, 2022). Imported Western technologies often exacerbate the global digital divide, highlighting the need for locally embedded tools to ensure equitable access (Alenazi, 2023; Alem, 2019; Aswad et al., 2022; Su et al., 2021). Despite their ubiquity, the instructional use of WeChat and Tencent Meeting for IELTS writing remains highly fragmented, focused on singular informal activities, and lacking cohesive theoretical grounding or alignment with official IELTS assessment criteria (Luo et al., 2023; Xie et al., 2022). Second, existing TELL research for high-stakes IELTS writing has long isolated writing performance outcomes from user acceptance—a core, evidence-based predictor of sustained tool use and long-term learning gains—creating a critical disconnect between intervention efficacy and real-world sustainability (Fitria et al., 2024; Venkatesh et al., 2012). Third, Third, the motivational and affective benefits of technology-mediated writing interventions remain severely underdiscussed in Asia-Pacific EFL contexts, despite being core mechanisms for long-term outcomes.

Theoretically, three established frameworks provide the synergistic foundation for this study’s integrated intervention design. First, Web-Based Cognitive Writing Instruction (WeCWI) offers a validated pedagogical framework for fostering ESL/EFL learners’ academic writing, critical thinking, and self-regulated learning through a structured “Read→Write→Discuss” cycle, with its efficacy well-documented in prior EFL writing research (Mah, 2022, 2023; Mah and Cheah, 2022; Mah et al., 2021). Second, the ADDIE (Analysis, Design, Development, Implementation, Evaluation) model provides a systematic, standardized backbone for context-adapted instructional tool development, ensuring rigorous, learner-centered intervention design (Branch and Merrill, 2012; Gamal, 2022). Third, the Unified Theory of Acceptance and Use of Technology (UTAUT) identifies four core determinants of technology adoption—Performance Expectancy, Effort Expectancy, Social Influence, and Facilitating Conditions—that explain 70% of variance in user acceptance, with its adaptations for voluntary and learner-centered educational settings (Bessadok and Hersi, 2025; Nguyen and Chu, 2021; Venkatesh et al., 2003; Venkatesh et al., 2012;). Critically, the majority of UTAUT investigations in EFL have focused exclusively on user perception and behavioral intention, without linking acceptance to actual learning performance data, while overlooking the framework’s direct conceptual links to learners’ affective and motivational states (Hsu, 2023; Fitria et al., 2024;). These frameworks are further grounded in foundational second language acquisition (SLA) theory, including Schmidt’s (1990) noticing hypothesis, Long’s (1996) interaction hypothesis, and Swain’s (1995) output hypothesis, which collectively emphasize attention, interaction, and targeted output as critical to L2 writing development, alongside Hyland’s (2019) seminal work on second language writing pedagogy.

Existing research has yet to address three synthesized gaps. First, traditional instruction fails to resolve persistent IELTS writing difficulties (Arzhadeeva and Kudinova, 2020; Buitrago and Díaz, 2017; Jafary et al., 2023; Limpo et al., 2020; Panahi and Mohammaditabar, 2015; Truong, 2022; Wicaksono et al., 2023), and empirically validated, context-appropriate TELL tools remain scarce (Braun and Clarke, 2006; Chen et al., 2025; Polit et al., 2007; Qin et al., 2022; Suo et al., 2025a; Wang, 2023). Second, local platforms like WeChat and Tencent Meeting are used fragmentally, lacking systematic integration of frameworks like WeCWI, ADDIE, and UTAUT to align tool design with pedagogical logic (Alasmari and Zhang, 2019). Third, TELL research frequently isolates writing performance from user acceptance and underdiscusses the affective-motivational benefits of these interventions.

**Figure 1:**
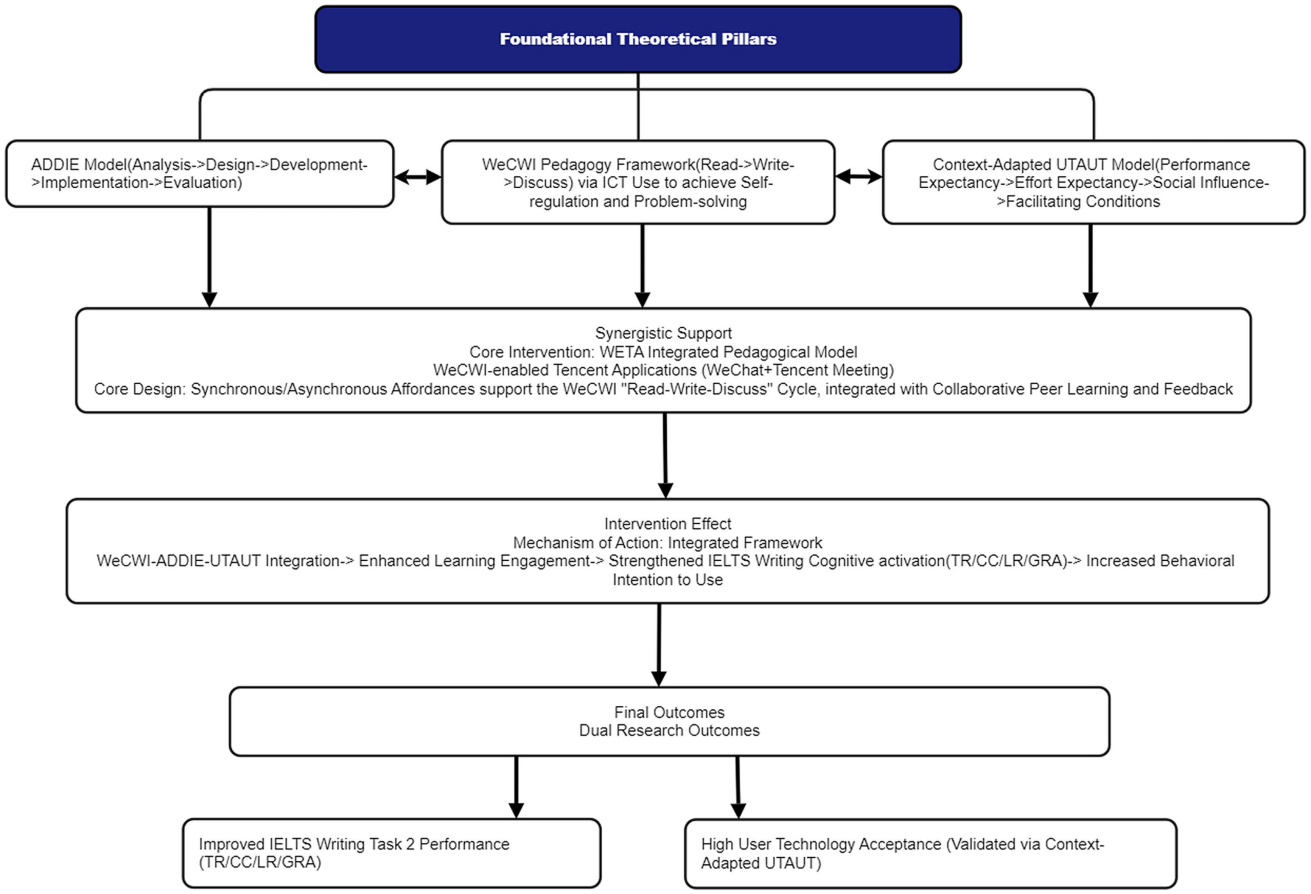
The theoretical framework of the WETA integrated pedagogical model.

To address these gaps, this study designed, implemented, and assessed the WeCWI-Enabled Tencent Applications (WETA), a blended TELL tool integrating WeChat, Tencent Meeting, and the three-pillar WeCWI-ADDIE-UTAUT framework. This integrated framework and its underlying mechanism are visually depicted in Figure 1, thereby linking the intervention to increased learning engagement, potentially reduced affective barriers, and better learning outcomes. Using an explanatory sequential mixed-methods design, 60 Chinese EFL undergraduates were split into an 8-week WETA intervention/experimental group (*n* = 30) and a traditional control group (*n* = 30). This study investigates three objectives: (1) examining WETA’s effects on IELTS Writing Task 2 performance; (2) investigating user acceptance via the UTAUT framework; and (3) uncovering the intervention’s implied motivational and affective benefits.

Accordingly, three core research questions guide this study:

1. Is the WETA intervention significantly associated with improvements in Chinese EFL learners’ IELTS Writing Task 2 performance?
2. Is user acceptance of WETA supported by the UTAUT framework?
3. What are the implied motivational and affective benefits of the WETA intervention for learners’ writing-related experiences?

Two hypotheses were tested:

*H_1_*: The WETA intervention group will demonstrate statistically significantly higher post-test scores in IELTS Writing Task 2 overall scores and sub-criteria scores (TR, CC, LR, GRA) compared to the control group (traditional instruction) after the eight-week intervention.

*H_0_*: There will be no statistically significant difference in IELTS Writing Task 2 overall scores or sub-criteria scores (TR, CC, LR, GRA) between the WETA intervention group and the control group after the eight-week intervention.

This study makes three distinct contributions to TELL and educational psychology research. Theoretically, it demonstrates the feasibility of the first integrated WeCWI-ADDIE-UTAUT framework for non-Western TELL contexts, extends WeCWI to high-stakes IELTS preparation, and refines the Western-centric UTAUT model to explore potential affective-motivational associations in collectivist Asian EFL settings. Practically, it provides a low-cost, context-aligned TELL tool that is associated with gains in writing proficiency, delivers high user acceptance, and supports learners’ psychological well-being in high-pressure exam environments. Globally, it illustrates potential alignment with UN SDG 4 (Quality Education) by offering a theoretically scalable, equity-focused TELL model for resource-constrained contexts. This suggests a pathway to address the educational digital divide without reliance on expensive Western platforms (Alenazi, 2023; UNESCO, 2023).

## 2 Materials and methods

### 2.1 Research design

To address the above-mentioned three gaps, this study designed the WETA intervention and employed an explanatory sequential mixed-methods design (Creswell and Clark, 2017). The dominant quantitative phase utilized a quasi-experiment to assess WETA’s effects on IELTS Writing Task 2 performance. The subsequent qualitative phase involved semi-structured interviews to investigate user acceptance and implied affective-motivational benefits based on the UTAUT model. This sequential approach aligns with the ADDIE model’s Implementation and Evaluation phases, allowing qualitative data to explain and validate quantitative outcomes, thereby creating a cohesive evaluation process (Johnson and Onwuegbuzie, 2004).

### 2.2 Participants

Participants in this study were Chinese EFL university undergraduates (aged 21–25) from the English Department of Beijing Union University, and who had previous IELTS training or testing experience. Criterion sampling was used whereby 60 participants were selected; 30 in the intervention Group (EG; WETA-supported instruction) and 30 in the Control Group (CG; traditional classroom instruction). In order to eliminate selection bias, the two groups were created from intact classes and a pretest (IELTS Writing Task 2) and a demographic questionnaire (gender, IELTS experience, English proficiency) were administered to ensure baseline equivalence.

Patton (2015) emphasizes the importance of diversity in purposeful sampling to capture varied experiences; accordingly, to ensure diversity in user acceptance perspectives. For the qualitative phase, six students (high, medium, low = 2 each) from the intervention group are purposefully selected, with an IELTS writing instructor (who teaches both groups) invited to prove the feasibility of WETA in the future teaching and learning. In order to assure the saturation and representativeness of the six classified student participants and one senior instructor, two students and one teacher of them were recruited for saturation testing, with no new conceptual categories or coding nodes identified from their responses.

Using G*Power 3.1, a power analysis was done to establish the appropriate sample size. Having determined *α* = 0.05, Power = 0.8, and expected effect size *f* = 0.4 focusing on ANCOVA as the core analytical approach, the minimum sample size was 26 participants per group. Considering that this study involved 30 participants from each group (60 participants total), it meant that the statistical power requirements, as well as the research result’s robustness, was met.

### 2.3 Intervention: WETA

The 8-week WETA intervention (2 h weekly) was built on WeCWI pedagogy and the ADDIE model. It merges synchronous delivery on Tencent Meeting and asynchronous interaction on WeChat. It is made up of 8 teaching modules which map to the four IELTS Writing Task 2 assessment criteria, and Second Language Acquisition (SLA) theory. The core components include: (1) Instruction via Tencent Meeting (analytical modeling of essays, discussions in breakout rooms, collaborative brainstorming); (2) Interaction via WeChat (resource sharing, feedback sharing); (3) Reading-writing-collaborative learning activities aligned to WeCWI; (4) Targeted practice related to IELTS criteria; (5) A dual teacher-peer feedback aligned with official writing rubrics. The intervention was implemented with full fidelity. The 2-h weekly session was structured as a blended learning period, comprising both synchronous instruction via Tencent Meeting and asynchronous interaction via WeChat within the allocated 2 h. Rather than adding extra instructional hours, the WeChat-based activities (e.g., resource sharing, peer feedback) were integrated into the session to replace traditional in-class textbook exercises. Student engagement was tracked through post activity metrics which included reading, writing, discussion, and feedback.

The CG received identical instructional content and contact hours (2 h weekly) via traditional teacher-led lectures, textbook practice, and paper assignments, with no WETA integration. Out-of-class assignments (e.g., essay drafting) were identical in scope and estimated workload for both groups to ensure no dosage effects confounded the intervention. WETA’s design centers on educational equity, addressing digital support disparities across educational contexts (Balcı, 2024; Irgatoglu, 2021). Built on widely accessible, low-technical-barrier Tencent platforms, it minimizes skill training needs and costs for students across social strata (Luo et al., 2023; Wang and Luo, 2022), aligning with equitable edtech design principles that avoid widening resource inequities (Alenazi, 2023; Alem, 2019; Masoud, 2024). 24/7 WeChat-based technical support was provided to remove learning barriers (Fan et al., 2023; Qin et al., 2022).

To uphold intervention fidelity, both groups were taught by the same IELTS instructor with over 5 years of teaching experience, who completed 2 weeks of standardized training to ensure content consistency. Two independent raters verified 30% of course recordings via a fidelity checklist, confirming over 95% content consistency between groups, confirming the intervention effect was driven by WETA rather than teaching differences.

To account for any possible instructor effects, both interventions were conducted by a certified IELTS instructor with standardized protocols, and pre-test scores were used as the covariate in the ANCOVA for baseline equivalence, which is a standard approach in quasi-experimental educational research.

#### 2.3.1 WETA’s mechanism for writing improvement

The WETA intervention is grounded in the synergistic integration of three frameworks: WeCWI provides the pedagogical logic (Read→Write→Discuss), the ADDIE model structures the systematic implementation process, and UTAUT informs the evaluation design to ensure high user acceptance. WETA’s effectiveness operates through two mutually reinforcing mechanisms that align with this integrated design logic.

WETA’s association with improved IELTS Writing Task 2 performance is interpreted through the integrated theoretical framework proposed in this study (ADDIE + WeCWI pedagogy + contextualized UTAUT), and operates through two mutually reinforcing mechanisms that align with its design logic and functional structure.

First, systematic cognitive activation driven by WeCWI’s core tasks. As the foundational pedagogical core of WETA, WeCWI’s reading–writing–discussion cycle via ICT use to achieve self-regulation and problem-solving is fully operationalized to target the four IELTS assessment criteria. Through synchronous activities in Tencent Meeting (e.g., model essay analysis, collaborative brainstorming, and real-time discussion) and asynchronous interaction in WeChat (e.g., resource sharing, self-paced review, and peer feedback), learners engage in deep cognitive processing: reading and collaboratively analyzing authentic IELTS materials through collaborative discussion and peer feedback associated with strengthening Task Response (TR); discussing and co-constructing logical structures linked to enhanced Coherence and Cohesion (CC); sharing and practicing topic-specific lexical items correlating with enriched Lexical Resources (LR); and receiving focused feedback on grammatical forms associated with improvements in Grammatical Range and Accuracy (GRA). Such actions also adheres to the systematic design procedure of the ADDIE model, ensuring that every learning activity is intentionally structured in relation to scoring rubrics and learner gaps.

Second, the contextualized synergy of synchronous–asynchronous interaction and dual-path feedback. WETA merges the face-to-face communication (synchronous) of Tencent Meeting and the messaging (asynchronous) of WeChat to facilitate uninterrupted engagement in learning. In addition, interactive instructional feedback and peer feedback is integrated. This collaborative feedback system cognitive load and learning barriers and reinforces the three fundamental UTAUT constructs of effort expectancy, social influence, and facilitating conditions. Especially, low Effort Expectancy is associated with reduced cognitive load and perceived technostress, potentially creating conditions that may alleviate technology-related friction. Meanwhile, Social Influence through peer collaboration fosters a supportive environment theoretically linked to increased self-efficacy, though it must be noted that UTAUT constructs measure user acceptance rather than motivational-affective states directly. As a result of contextual adaptation, the technology is not a disjointed technical adjunct, but rather, accessible, acceptable, and sustainable to Chinese EFL students.

Particularly, the improvements in four IELTS scoring sub-criteria were a result of cognitive core activation of “read, write, and discuss,” as well as interaction and feedback mechanisms, especially, synchronous-asynchronous, and targeted feedback. Writing enhancement resulted from the structured cognitive activation of the WeCWI cycles and the integrated direct skill support (real-time feedback, WeChat, and error correction) in WETA’s instructional design (Mah et al., 2021).

Together, these mechanisms reflect a “cognitive-contextual synergy,” integrating localized technology and contextualized learning support from the theoretical framework which is associated with significant and measurable gains in IELTS Writing Task 2.

### 2.4 Instruments

#### 2.4.1 IELTS Writing Task 2 analytic rubrics

Essays from pretests and posttests are evaluated with the IELTS Writing Task 2 analytic rubrics (IELTS, n.d.). Each rubric of its four IELTS scoring sub-criteria is scored on a 9-band scale, with overall scores calculated as an average of the four sub-scores. Essays are scored by two independent raters (IELTS-certified instructors with more than 5 years of experience). Inter-rater reliability is established using Cohen’s kappa (target *κ* ≥ 0.70) (McHugh, 2012). Discussion is used to resolve discrepancies.

A same-prompt test–retest design was implemented for both the pretest and posttest. Administering an identical writing topic across the two assessment points mitigated potential confounding variance in background knowledge that a topic alteration might introduce over the eight-week interval. The selected prompt was evaluated against three methodological criteria. First, genre alignment was ensured, as the prompt required an argumentative essay of the opinion/discuss type, corresponding to the most frequent IELTS Task 2 format. Second, cognitive demand was considered; the prompt addressed an abstract social issue necessitating higher-order cognitive skills such as evaluation and argumentation rather than mere description. Third, regarding topic provenance, the prompt was sourced from official Cambridge IELTS past materials, aligning with authentic IELTS Writing Task 2 prompts cataloged by Suo (2019). Because the identical prompt was utilized for both measurements, the objective was to ascertain that the topic’s cognitive load remained appropriate for the participants, rather than to establish psychometric parallel-form equivalence between distinct tasks. The exact prompt is delineated in Appendix A.

#### 2.4.2 Semi-structured interview protocols

Based on the UTAUT framework, separate, distinct interview protocols are created for the students and the instructor. In the student interviews, questions address performance expectancy (e.g., “How has WETA helped improve your IELTS writing?”), effort expectancy (e.g., “How easy was it to use WETA’s features?”), social influence (e.g., “Did peers/teachers influence your use of WETA?”), and facilitating conditions (e.g., “Did you have adequate technical support for WETA?”). The instructor interview protocol has additional questions about pedagogical alignment and challenges of implementation. Each interview is recorded and transcribed as is.

To operationalize the contextually adapted UTAUT constructs for Chinese EFL contexts, the interview protocols were designed with differentiated coding dimensions aligned with the revised constructs. For performance expectancy, coding dimensions included perceived improvements in IELTS writing sub-criteria and task-specific skill development; for effort expectancy, dimensions focused on compatibility with Tencent ecosystem habits and tool learning duration; for social influence, dimensions encompassed teacher authority (instructor guidance and recommendation) and collective peer motivation (group collaboration and peer feedback); for facilitating conditions, dimensions included localized digital infrastructure support and 24/7 technical assistance via WeChat groups.

#### 2.4.3 Instrument validation

As stated by Suo et al. (2025a), the WETA tool underwent rigorous content validation, with the complete validation and raw data process being available in the peer-reviewed publication. Four experts (two IELTS certified with over a decade of teaching experience and two TELL researchers focusing on ICT integrated language teaching) reviewed WETA through a 27-item mixed-method (4-point Likert items and qualitative feedback) across five areas: content, design and usability, technology, pedagogy, and instructional and assessment materials. They agreed that both item-level content validity index (I-CVI) and scale-level content validity index (S-CVI/Ave) reached 1.0, which is well above the accepted benchmark of I-CVI ≥ 0.78 and S-CVI ≥ 0.90 (Polit et al., 2007). Also, for validation, acceptable internal consistency of the checklist (Cronbach’s alpha = 0.681) and strong inter-coder reliability for theme analysis of open-ended comments (Cohen’s *κ* = 0.78; Braun and Clarke, 2006) is additional evidence for the thoroughness of the validation process. Suo et al. (2025a) detail how revisions aimed at increasing usability and theoretical fit with the WeCWI framework were incorporated through the use of expert feedback. These revisions enhanced WETA’s “topic matrix” function (i.e., theme-based curriculum framework incorporating IELTS high-frequency topics and assessment criteria and WeCWI learning activities).

### 2.5 Data collection procedures

1. Both groups do an IELTS Writing Task 2 pretest (40 min) to establish baseline performance. Demographic questionnaires were assigned and administrated to collect participant characteristics.
2. The EG is given the eight-week WETA intervention. The CG receives standard instruction. All sessions (e.g., Tencent Meeting recordings, WeChat chat logs) were recorded to maintain fidelity.
3. After the intervention, both groups will do an IELTS Writing Task 2 posttest (40 min) using a prompt of similar difficulty and format as the pretest.
4. Semi-structured interviews with six students (30–40 min each) and one instructor (40–50 min) were conducted within 1 week after the posttest.

Qualitative data were collected via semi-structured focus group interviews with 6 stratified students (stratified by pre-test writing performance) and 1 individual instructor interview, all conducted in Mandarin to ensure natural expression. Each student focus group interview lasted 30–40 min and the instructor interview lasted 40–50 min, with a semi-structured interview guide developed based on the adapted UTAUT constructs; member checking was conducted after transcription, where all participants reviewed and confirmed the accuracy of the interview transcripts to ensure the authenticity of their perceptions.

### 2.6 Data analysis

#### 2.6.1 Quantitative analysis

Data were analyzed quantitatively using the software package SPSS version 29.0.

1. Independent samples t-tests were conducted to establish baseline equivalence by comparing pretest results from the EG and the CG.
2. Paired samples *t*-tests were used to analyze changes within groups from pretest to posttest.
3. One-way ANCOVA was employed to compare posttest scores between groups, with pretest scores as covariates to control for baseline differences.

#### 2.6.2 Qualitative analysis

Qualitative data were derived from semi-structured interviews with 6 stratified students and 1 instructor, analyzed through a thematic coding approach guided by the contextually adapted UTAUT framework (Venkatesh et al., 2003). Deductive coding was first performed based on the four core constructs of the context-adapted UTAUT framework, with some emergent coding selected to reflect learners’ writing-related motivational and affective experiences. The UTAUT-based coding manual, with differentiated dimensions for student (tool use experience) and instructor (pedagogical alignment, implementation challenges) interviews, was validated by two SLA/TELL experts. Two researchers independently executed three-stage coding—comprising open, axial, and selective phases—resulting in an interrater Cohen’s *κ* of 0.67 and a Spearman-Brown coefficient of *R* = 0.911. While this κ value marginally falls below the 0.70 threshold frequently cited for quantitative research, interpretive thematic analysis operates under different parameters. Landis and Koch (1977) classify κ values between 0.61 and 0.80 as indicating ‘substantial agreement.’ This range is widely deemed acceptable within qualitative coding frameworks, where researchers negotiate meaning around emergent, text-bound themes rather than categorizing items into mutually exclusive fixed variables. Discrepancies identified during the coding process were resolved through collaborative discussion, culminating in a reliable and converged final dataset.

Data saturation was rigorously verified: no novel codes emerged after coding the 5th transcript, and three supplementary interviews (2 students, 1 teacher) yielded no new coding nodes, confirming theoretical saturation (Charmaz, 2014). All interview data and ANCOVA analysis materials were derived from the author’s doctoral research (Suo, 2026). Two TELL/UTAUT experts completed independent double-checking of coding results, with 100% consistency in UTAUT construct alignment. Qualitative perceptions from the 6 students were triangulated with quantitative data (pre/post-test score gains, writing challenge surveys) from all 30 experimental group students, verifying the findings’ representativeness and external validity.

### 2.7 Ethical considerations

Ethical approval was granted by the Research Ethics Committee of Universiti Teknologi MARA (Malaysia) with the REC code, and institutional approval was obtained from Beijing Union University (China) prior to data collection. All participants provided written informed consent and the right to withdraw at any time without penalty. All data were anonymized and stored securely on a password-protected server accessible only to the research team. Member checking was conducted to ensure qualitative data accuracy. To ensure educational equity, the CG received full access to WETA materials after the study. Instructions and interviews were available in Mandarin for participants with lower English proficiency. This study was conducted in accordance with the 1964 Declaration of Helsinki and its later amendments.

## 3 Results

**Figure 2:**
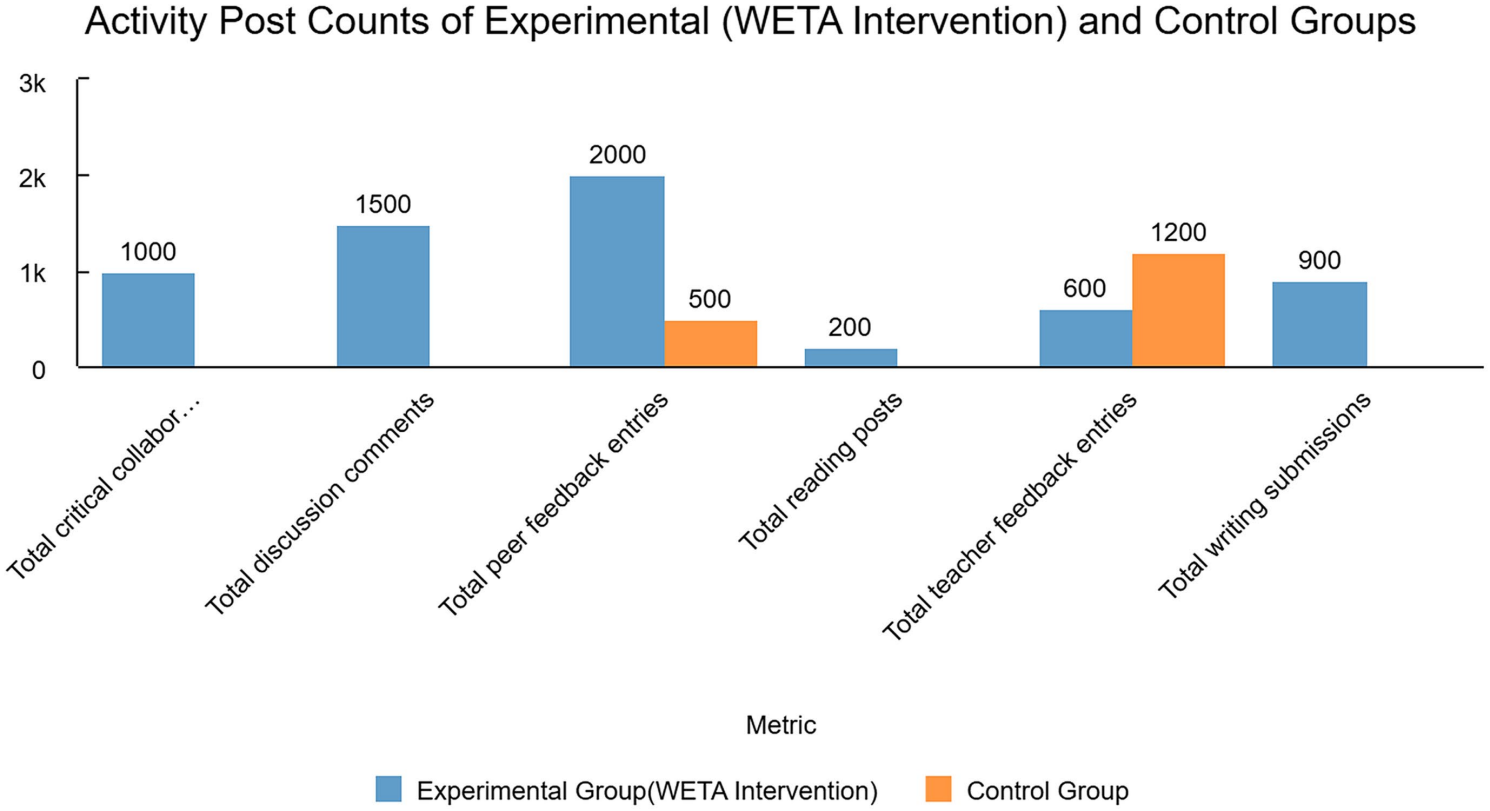
Activity post counts of experimental (WETA intervention) and control groups.

To ensure the rigor of the intervention and the validity of the main findings, preliminary analyses of student engagement (including activity post counts such as reading posts, writing submissions, discussion comments, and peer/teacher feedback entries) shown in Figure 2 were conducted prior to the main effect analyses. These preliminary analyses served to monitor intervention fidelity and student participation throughout the 8-week intervention period.

1. Activity engagement in the EG, including total reading posts, total writing submissions, total discussion comments, total critical collaborative reflections, total peer feedback entries, and total teacher feedback entries.
2. Activity engagement in the CG, where most interactive tasks were not applicable (zero counts).

As is manifest in Figure 2, the use of WETA was associated with increased learners’ engagement in their learning, with their activity post counts relevant to reading posts, writing submissions, discussion comments, and peer/teacher feedback entries being markedly higher than their counterparts in CG.

The section elaborates the data retrieved through quantitative analysis of a quasi-experimental study of a particular variable; so does the effect of the WETA intervention and how it affects Chinese EFL (English as a Foreign Language) undergraduates’ performances in the IELTS Writing Task 2 exam. This study has a total of 60 participants, which were subdivided into an EG and a CG (30 participants in each group). A summary and brief description of the main findings are given below.

### 3.1 Participants and baseline equivalence

The participants were EFL undergraduates, aged 21–25, with moderate English proficiency, as well as previous training and/or experience with the IELTS exam. Participants were selected from two university classes (non-random assignment consistent with quasi-experimental design; the gender ratio was not a research focus). In the pretest analysis, there were no major findings in the EG and CG groups in total scores of IELTS Writing Task 2, and in each of the four sub dimensions (Task Response [TR], Coherence and Cohesion [CC], Lexical Resources (LR) and Grammatical Range and accuracy [GRA]), thus establishing a no significant comparability of baseline writing performance between the two groups. Independent samples *t*-tests confirmed no significant differences between the EG and CG in key confounding variables, including English learning motivation (*t* = 0.87, *p* = 0.39), digital literacy (*t* = 0.92, *p* = 0.36), and prior IELTS learning duration (*t* = 0.75, *p* = 0.46), all *p* > 0.05. For the EG specifically, baseline data showed 23.3% (*n* = 7) of participants had low digital literacy, with the remaining 76.7% (*n* = 23) at medium or high levels, confirming a representative distribution of digital skill levels in the study sample.

### 3.2 Pretest-posttest performance changes

Table 1 presents the pretest and posttest scores for both groups. Pretest analyses via independent samples t-tests revealed no significant differences in overall IELTS Writing Task 2 performance or four sub-criteria between the EG and CG, establishing baseline equivalence. Results from the posttest (Table 1) showed the EG made notable gains in all areas (total score: 5.50 → 6.65; Lexical Resources: 5.43 → 6.63 as the most significant)—with gains noted across all EG participants no matter their level of digital skills, as the core operations of the intervention were mastered in about 15 min due to their integration with the participants’ existing digital tool usage, which illustrates WETA’s inclusive design that closes the potential digital divide in EFL learning. In contrast, the CG made only slight improvements (total score: 5.33 → 5.62). A descriptive subgroup analysis of the EG further corroborated consistent and statistically significant gains in writing across all levels of digital literacy, confirming WETA’s equal learning outcomes for learners with different levels of digital skills.

**Table 1:** Means and standard deviations of the pre-test and post-test.

Variables	Group	Pre-test		Post-test	
		Mean	S.D.	Mean	S.D.
Total scores	EG	5.50	0.44	6.65	0.37
	CG	5.33	0.44	5.62	0.34
Task response	EG	5.20	0.61	6.40	0.50
	CG	5.13	0.50	5.60	0.50
Coherence and cohesion	EG	5.50	0.57	6.53	0.57
	CG	5.27	0.52	5.53	0.63
Lexical resources	EG	5.43	0.57	6.63	0.56
	CG	5.20	0.61	5.33	0.66
Grammatical range and accuracy	EG	5.53	0.57	6.40	0.50
	CG	5.37	0.49	5.67	0.48

EG, experimental group (*N* = 30); CG, control group (*N* = 30).

### 3.3 Validation of ANCOVA assumptions

**Figure 3:**
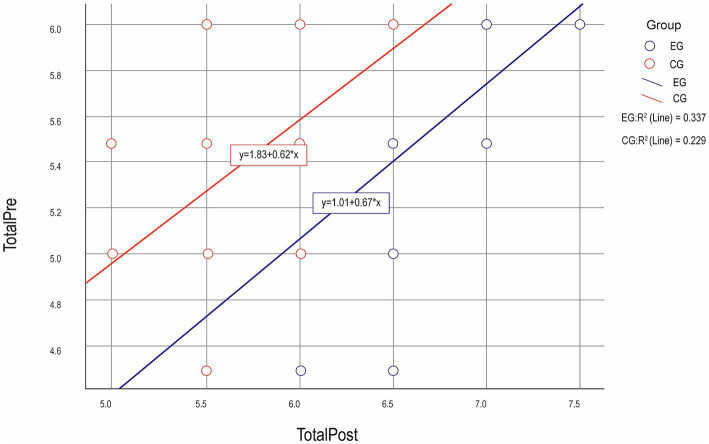
Scatter plot graph of post-test scores by pre-test scores for both experimental and control groups.

ANCOVA was utilized to determine the effect of the intervention while controlling for pretest scores as covariates, and all five primary assumptions of ANCOVA were completely met after thorough checks:

1. Normality: Skewness and kurtosis of all posttest variables for EG (Table 2) and CG (Table 3) were within the range of ±2 (Dunn, 1995; George and Mallery, 1999), confirming normality.
2. Linear relationship: The scatter plot (Figure 3) showed a linear relationship for the pretest (covariate) and posttest (dependent variable) scores for both groups, and with parallel slopes, the posttest score differences were attributable to the intervention.
3. Homogeneity of variances: Levene’s test (Table 4) yielded *p* > 0.05 for all variables, satisfying the assumption.
4. Homogeneity of regression slopes: Test between-subjects effects (Table 5) showed no significant pretest-group interaction (*p* > 0.05) for all dimensions.
5. Independence: Intact, non-overlapping groups with continuous dependent variable data; pretest scores and group assignment were independent (*t* = 1.12, *p* = 0.27).

**Table 2:** Skewness and kurtosis scores of the experimental group.

Variables		Total scores	Task response	Coherence and cohesion	Lexical resources	Grammatical range and accuracy
*N*	Valid	30	30	30	30	30
	Missing	0	0	0	0	0
	Mean	6.683	6.40	6.53	6.63	6.50
Skewness		0.897	0.430	0.456	−0.074	0.430
Std. Error of skewness		0.427	0.427	0.427	0.427	0.427
Kurtosis		0.769	−1.950	−0.748	−0.796	−1.950
Std. Error of kurtosis		0.833	0.833	0.833	0.833	0.833

**Table 3:** Skewness and kurtosis scores of the control group.

Variables		Total scores	Task response	Coherence and cohesion	Lexical resources	Grammatical range and accuracy
*N*	Valid	30	30	30	30	30
	Missing	0	0	0	0	0
	Mean	5.617	5.60	5.53	5.33	5.67
Skewness		0.385	−0.430	−0.133	−0.484	−0.745
Std. Error of skewness		0.427	0.427	0.427	0.427	0.427
Kurtosis		0.556	−1.950	−0.104	−0.620	−1.554
Std. Error of kurtosis		0.833	0.833	0.833	0.833	0.833

**Table 4:** Levene’s test of equality of error variances.

Variables	*F*	df1	df2	Sig.
Total scores	0.821	1	58	0.369
Task response	0.281	1	58	0.598
Coherence and cohesion	3.974	1	58	0.051
Lexical resources	3.890	1	58	0.053
Grammatical range and accuracy	2.236	1	58	0.140

**Table 5:** Results of test between-subjects effects.

Variables	Type III sum of squares	df	Mean square	*F*	Sig.
Group*Total scores	0.049	1	0.049	0.519	0.474
Group*Task response	0.098	1	0.098	0.478	0.492
Group*Coherence and cohesion	0.106	1	0.106	0.386	0.537
Group*Lexical resources	0.526	1	0.526	1.563	0.216
Group*Grammatical range and accuracy	0.175	1	0.175	1.028	0.315

One-way ANCOVA results (Table 6) indicated significant differences in posttest total scores (*F* = 143.017, *p* < 0.001, partial *η*^2^ = 0.715) and all four subdimensions (*p* < 0.001) between EG and CG, with all partial *η*^2^ values exceeding 0.138 (Cohen, 1988) indicating large effect sizes. WETA intervention significantly improved EG’s performance: total score (adjusted mean: 6.134 vs. CG’s 5.647), Lexical Resources (6.594 vs. 5.372) and Coherence and Cohesion (6.468 vs. 5.599) showed the most prominent gains.

**Table 6:** Results of one-way ANCOVA tests.

Variables	Group	*N*	Mean	S.D.	Adjusted mean	S.E.	*F*	*p*	Partial *η*^2^
Total scores	EG	30	6.650	0.375	6.134	0.056	143.017	<0.001	0.715
	CG	30	5.617	0.339	5.647	0.056			
Task response	EG	30	6.40	0.498	6.387	0.082	44.108	<0.001	0.436
	CG	30	5.60	0.498	5.613	0.082			
Coherence and cohesion	EG	30	6.53	0.571	6.468	0.096	39.862	<0.001	0.412
	CG	30	5.53	0.629	5.599	0.096			
Lexical resources	EG	30	6.63	0.556	6.594	0.107	63.342	<0.001	0.526
	CG	30	5.53	0.661	5.372	0.107			
Grammatical range and accuracy	EG	30	6.40	0.498	6.358	0.076	36.233	<0.001	0.389
	CG	30	5.67	0.479	5.709	0.076			

EG, experimental group; CG, control group.

Reliability of pretest and posttest scores (rated by two experienced teachers) was assessed via ICC (two-way mixed effects model).

Table 7 showed ICC values ranged from 0.894 to 0.986, representing good to excellent reliability (Koo and Li, 2016).

**Table 7:** Reliability statistics for the pre-test and post-test scores.

Dimensions	Intraclass correlation	*p*
Pre-test total scores	0.894	<0.001
Task response	0.975	<0.001
Coherence and cohesion	0.951	<0.001
Lexical resources	0.960	<0.001
Grammatical range and accuracy	0.950	<0.001
Post-test total scores	0.970	<0.001
Task response	0.971	<0.001
Coherence and cohesion	0.984	<0.001
Lexical resources	0.986	<0.001
Grammatical range and accuracy	0.969	<0.001

Construct validity was verified via KMO and Bartlett’s Test (Table 8): pretest KMO = 0.728, posttest KMO = 0.749 (both >0.5, supporting factor analysis); Bartlett’s Test yielded χ^2^ = 52.426 (pretest, df = 6, *p* < 0.001) and χ^2^ = 79.750 (posttest, df = 6, *p* < 0.001), rejecting the null hypothesis and confirming significant intercorrelations among subdimensions. The test questions were response-validated based on four experts’ review and alignment to the official IELTS band descriptors.

**Table 8:** KMO values and Bartlett’s test.

Module name	Chi square	df	Pre-test	Chi square	df	Post-test
KMO value			0.728			0.749
Bartlett’s test	52.426	6	0.000	79.750	6	0.000

To summarize, the WETA intervention was associated with significant improvements in Chinese EFL undergraduates’ IELTS Writing Task Two across all measured dimensions. The effect sizes prove the intervention’s pedagogical value. Targeted improvements were evident in the WETA EG’s IELTS Writing Task 2 scores and all subset criteria, when compared to CG. Thus, Hypothesis H_1_ is confirmed, and null hypothesis H_0_ is invalid.

### 3.4 User acceptance of WETA (UTAUT-based findings)

**Figure 4:**
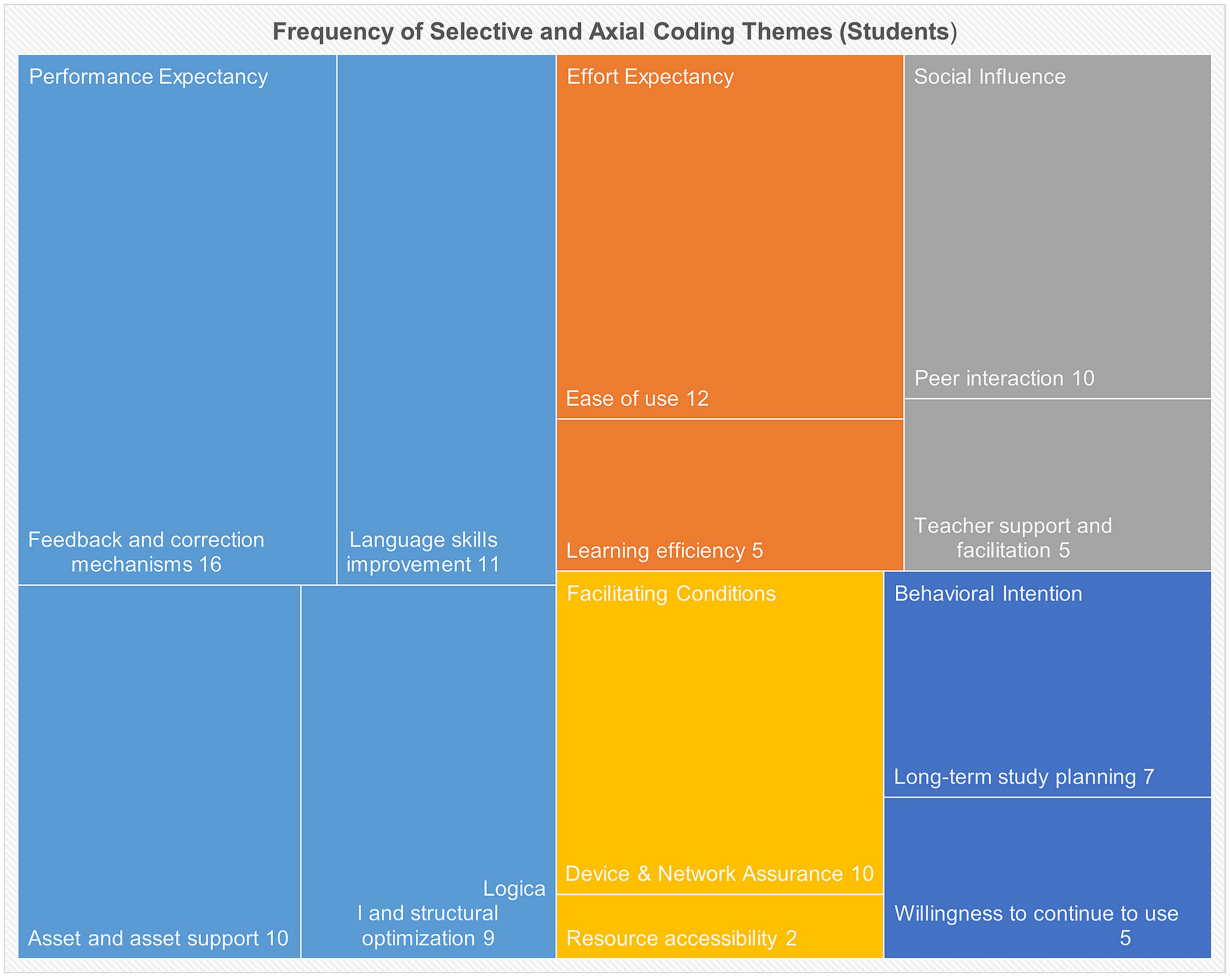
Frequency of selective and axial coding themes (students).

**Figure 5:**
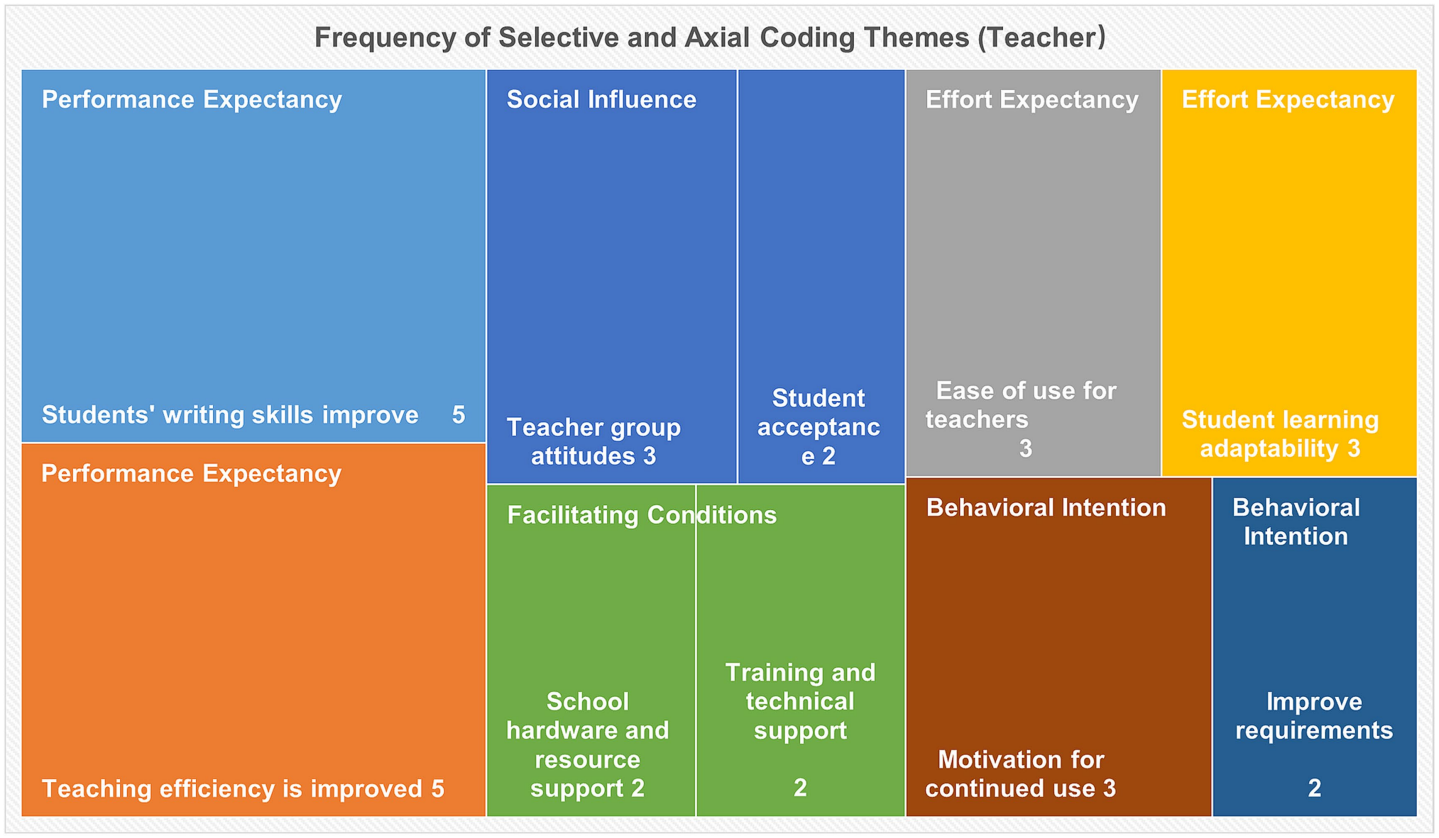
Frequency of selective and axial coding themes (teacher).

Based on the interview protocols of an IELTS instructor, semi-structured interviews were conducted, resulting in 7 interviews total. There were 6 stratified students involved in the study. They were stratified into three groups (high, medium, and low achievers), making it 2 students each. Qualitative data were analyzed using NVivo 14. Coding was done in three stages. For the first stage, open coding was employed; for the second, axial coding was applied; for the third and final stage, selective coding was adopted. UTAUT was the framework guiding the coding. For this study, thematic analysis was based on Corbin and Strauss (2014). A condensed overview of the coding metrics, hierarchical themes was presented in Figures 4, 5.

Open coding generated 34 initial reference nodes from student interviews and 24 from the instructor interview. For the student data, axial coding further refined the 34 nodes into 12 sub-themes, and final selective coding extracted 5 core themes that fully mapped to the UTAUT model’s four core constructs and behavioral intention (Venkatesh et al., 2003). The instructor data were processed separately; the 24 initial nodes were refined into 10 sub-themes, which were analyzed specifically for implementation themes rather than user acceptance (detailed in Section 3.4.6 and Figure 5). Rigorous reliability was confirmed with inter-rater consistency: Cohen’s *K* = 0.67 and a Spearman-Brown coefficient of *R* = 0.911. Theoretical saturation was verified via three supplementary interviews with no new coding nodes emerging, and external validity was ensured through triangulation with quantitative performance data from all 30 EG students. The following analysis aligns core coding themes with quasi-experimental ANCOVA results, with 1–2 paraphrased quotes per construct to support selective coding findings.

#### 3.4.1 Performance expectancy

Selective coding identified Performance Expectancy as the dominant driver of user acceptance, evidenced by 46 nodes derived exclusively from student interview – the highest coding frequency across all constructs. For the learners’ interview, axial coding linked similar open codes into four main sub-categories: asset and resource support (10 nodes), feedback and correction mechanisms (16 nodes), language skills development (11 nodes), and logical and structural improvement (9 nodes), associated with original open codes including vocabulary diversity optimization, error diagnosis, model essay benchmarking, and multi-dimensional argument expansion. Average learners appreciated the use of synonyms and collocations in the tool and breaking the cycle of repetitive vocabulary, whereas below average learners appreciated the visual error diagnosis in the tool and breaking the cycle of grammatical errors. The qualitative data directly supports the outcome of ANCOVA analysis. The analysis revealed significance across all four IELTS writing components and indicated a large effect size. In the Lexical Resources (LR) component, which had the most coding, the participants recorded the highest level of improvement (5.43 → 6.63, *p* < 0.001, partial *η*^2^ = 0.526). This was followed by the Task Response (TR) (5.20 → 6.40, partial *η*^2^ = 0.436), Cohesion and Coherence (CC) (5.50 → 6.53, partial *η*^2^ = 0.412), and Grammar Range and Accuracy (GRA) (5.53 → 6.40, partial *η*^2^ = 0.389) components.

#### 3.4.2 Effort expectancy

Selective coding categorized Effort Expectancy as the second most prominent construct, consisting of 17 nodes from students’ interviews. As for the students’ interviews, axial coding grouped the open codes into two main sub-categories: ease of use (12 nodes) and learning efficiency (5 nodes), which corresponds to the initial open codes of being a burden-free operation, quick internalization of functions, and easy interaction through a simplified interface. There were minimal learning barriers due to WETA’s integration with the daily-used Tencent ecosystem: medium-performing learners were able to master core functions after 15 min of initial guidance, and after 20 min of teacher training, high-performing students were able to complete the writing task independently. This perceived low effort was directly linked to the high engagement metrics in the EG and magnified the writing performance gains shown in the quantitative analysis.

#### 3.4.3 Social influence

Selective coding mapped Social Influence to 15 nodes from students’ interviews. Concerning student interviews, axial coding divided open codes into two categories: peer interaction and motivation (10 nodes) and teacher support and facilitation (5 nodes). These categories were based on the initial open codes of experience-sharing networks, group monitoring mechanisms, and resource collaborative teaching resource sharing. The interviews indicated that high achievers were driven by peer progress monitoring and co-feedback, whereas the low achievers pointed out that the favorable group environment and the differentiated feedback from the teacher were instrumental in making their writing improvement visible. This social reinforcement was the main driver for sustained involvement in the WETA collaborative learning activities, resulting in the EG significantly outperforming the CG in terms of participation in discussions and peer feedback, which was also linked to substantial improvements in writing.

#### 3.4.4 Facilitating conditions

Selective coding identified Facilitating Conditions with 12 nodes from students’ interviews. Regarding the interviews on students, axial coding grouped open codes into two sub-categories: Device and Network Assurance (10 nodes), and Resource Accessibility (2 nodes). These sub-categories corresponded to the initial open codes: multi- terminal compatibility, network stability, prompt technical response, offline caching policies, etc. WETA was fully compatible with school desktops and personal mobile devices, and 24/7 WeChat-based technical support led to quick resolution of operational problems, while offline caching eliminated disruptions caused by unstable networks. These strong facilitating conditions eliminated practical learning obstacles and ensured consistent engagement across all levels of digital literacy, as confirmed by subgroup analysis, which showed similar writing gains to students with low and high digital literacy.

#### 3.4.5 Behavioral intention

Twelve nodes from students out of seventeen nodes’ interviews detail Behavioral Intention to continue WETA use captured through selective coding. For the student interviews, concerning the student interviews, axial coding organized the open codes into two subcategories: long-term study planning (7 nodes) and willingness to continue to use (5 nodes) related to the open codes of exam performance optimization, advanced feature deep dive, and purpose-driven learning confidence. Students’ interviews revealed that high- and medium-performing students uniformly reported their intent to continue using WETA. This behavioral intention was synergistically driven by the four core UTAUT constructs: high Performance Expectancy offered a core motivation, low Effort Expectancy reduced adoption barriers, affirmative Social Influence increased the motivation and strong Facilitating Conditions removed the practical obstacles.

#### 3.4.6 Instructor perspectives: implementation feasibility and pedagogical alignment

To strictly avoid conflation of student acceptance with instructor evaluation, the instructor’s qualitative data are reported independently here. The instructor’s responses to the UTAUT-based interview do not measure student acceptance. Conversely, they provide critical insights from the implementer’s perspective on the operational and pedagogical dimensions of the WETA framework. Specifically, while the instructor’s pedagogical actions—such as streamlined feedback and active monitoring—constitute the objective implementation of Facilitating Conditions (FC) and Social Influence (SI) in the classroom, these reports confirm only that the intervention was delivered as designed, not how students subjectively perceived it.

The instructor’s perspective provides direct evidence of the implementation feasibility and pedagogical alignment of the WETA framework, identifying three core themes: (1) Streamlined feedback delivery (the dual teacher-peer mechanism allowed the instructor to shift focus from lower-order to higher-order errors); (2) Enhanced student engagement monitoring (WeChat activity logs provided real-time tracking of participation, confirming operational viability); and (3) Implementation challenges (managing the synchronous-asynchronous workflow required significant initial curriculum development labor). Ultimately, these instructor-derived themes confirm the operational viability and pedagogical alignment of the WETA design, independent of students’ own acceptance reports.

#### 3.4.7 Descriptive mapping of localized UTAUT constructs

Qualitative thematic analysis synchronized the UTAUT constructs with two distinct dimensions: student user acceptance and instructor-reported implementation feasibility/pedagogical alignment. In NVivo 14, coding node counts were brought out for descriptive purposes to show the number of mentions of each contextualized UTAUT construct in the interviews, rather than being used for quantitative validation or a proxy for strength of perception.

**Figure 6:**
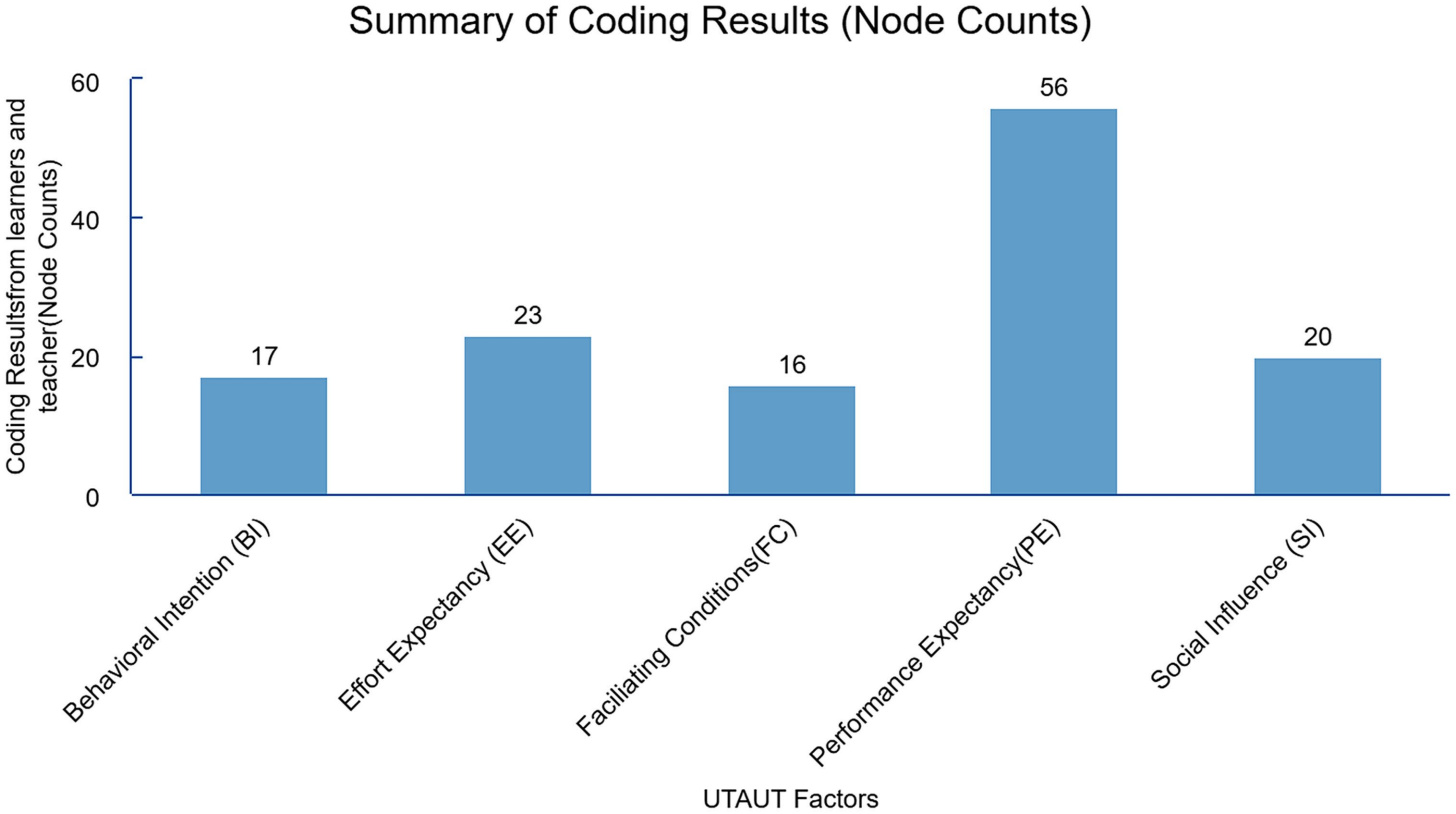
Summary of coding results (node counts).

As seen in Figures 4–6, student-reported nodes for the four primary constructs were high (Performance Expectancy: 46; Effort Expectancy: 17; Social Influence: 15; Facilitating Conditions: 12), with Behavioral Intention supported by 12 nodes, establishing the core user acceptance model. Concurrently, instructor-reported nodes focused on implementation themes (Performance Expectancy: 10; Effort Expectancy: 6; Social Influence: 5; Facilitating Conditions: 4). Descriptively, effort expectancy (compatibility of the Tencent ecosystem) and social influence (teacher authority and peer motivation) were the most prominent secondary drivers among students, which is coherent with the Chinese EFL context.

While instructor data are descriptively mapped alongside student data in the UTAUT framework (see Figures 4–6) to illustrate systemic coherence across the intervention design, it is crucial to emphasize that the core user acceptance model is driven solely by student-reported nodes; instructor nodes serve exclusively as indicators of implementation feasibility and pedagogical alignment, not as direct or indirect evidence of student acceptance.

### 3.5 Qualitative explanation for quantitative intervention effects

Three-stage coding results provide an elaborate, mechanism-based rationale for the substantial quantitative effect of the intervention in the quasi-experiment. The ANCOVA results indicated that the EG made statistically significant, large effect improvements in overall IELTS Writing Task 2 scores (5.50 → 6.65, *p* < 0.001, partial *η*^2^ = 0.715) and in all four sub-criteria as compared to CG’s negligible improvements (5.33 → 5.62). The qualitative coding results indicated that these improvements are directly related to the participants’ improvement in all four UTAUT dimensions: the strong Performance Expectancy stimulated vigorous involvement in self-directed peer learning, criterion-referenced learning, and assessment modules; the low Effort Expectancy reduced the cognitive load and increased the learning focus; the positive Social Influence extended the peer collaborative learning and sustained the collaborative learning; and the strong Facilitating Conditions eliminated the obstacles to the implementation of self-directed peer learning across all participant profiles.

The synergistic effect of these four constructs created a self-reinforcing cycle of engagement and skill development, further corroborated by the EG’s significantly higher activity engagement metrics (reading posts, writing submissions, feedback entries) compared to the CG. These findings suggest that UTAUT-driven user acceptance and feasibility of WETA was not merely a perceptual outcome, but a potential behavioral mechanism associated with the intervention’s substantial impact on IELTS writing performance improvements.

### 3.6 Implied motivational and affective benefits

Although this study did not employ standardized psychometric inventories to directly measure affective states, the mixed-methods data provide robust, multi-dimensional triangulation to address RQ3 regarding the implied motivational and affective benefits of the WETA intervention. These implied benefits are corroborated through three converging data sources: quantitative behavioral engagement, qualitative UTAUT-driven interview coding, and objective writing performance gains.

First, quantitative behavioral engagement metrics (Figure 2) imply a reduction in behavioral avoidance and potentially writing anxiety. The experimental group demonstrated significantly higher frequencies in out-of-class activity post counts—including reading posts, writing submissions, discussion comments, and peer/teacher feedback entries—compared to the control group. Such sustained, high-frequency interactive behaviors serve as objective indicators of active participation. This reduction in behavioral avoidance is theoretically aligned with a buffering effect against high-stakes writing anxiety, as learners who are typically apprehensive about writing tend to avoid engagement.

Second, qualitative reflections from the UTAUT-based interviews imply increased academic self-efficacy and reduced technostress. Within the Performance Expectancy construct, participants reported that visual error diagnosis and model essay benchmarking allowed them to break the cycle of repetitive vocabulary and grammatical errors, making their writing improvement visible. Such perceptions of mastery are theoretically linked to enhanced academic self-efficacy. Furthermore, within the Effort Expectancy construct, learners highlighted the “burden-free operation” due to WETA’s integration with the daily-used Tencent ecosystem, which was associated with reduced cognitive load and perceived technostress, implying a potential alleviation of technology-related friction. Additionally, the high Social Influence (peer interaction and teacher facilitation) created a supportive community of practice, further implying a catalytic effect on learning motivation.

Third, objective performance gains (ANCOVA results) provide an empirical anchor for the shift from apprehension to confident engagement. The significant and large-effect improvements in IELTS Writing Task 2 scores and all four sub-criteria (Table 6) bridge the gap between “perceived usefulness” and “empirical effectiveness.” This validated skill improvement provides the cognitive foundation for learners’ increased self-efficacy and potentially shifts their emotional trajectory from exam-driven apprehension to confident engagement.

Together, while the motivational-affective improvements remain ‘implied’ and hypothesis-generating in the absence of direct psychometric scaling, the convergent evidence of high user acceptance (UTAUT coding), high behavioral engagement (activity post counts), and objective performance gains provides a convergent theoretical support (via observed engagement, perceived acceptance, and performance gains) for the implied motivational-affective benefits, which remain hypothesis-generating pending direct psychometric validation.

## 4 Discussion and conclusion

### 4.1 Discussion of findings

The present quasi-experimental and mixed-methods findings indicate that WETA is associated with statistically significant, large-effect gains in IELTS Writing Task 2 performance and generates high UTAUT-driven user acceptance among Chinese EFL undergraduates, correlating with higher performance compared to traditional teacher-centered instruction. Unlike prior fragmented applications of WeChat and Tencent Meeting for EFL writing (Xie et al., 2022), WETA integrates WeCWI pedagogy, the ADDIE design model, and context-adapted UTAUT evaluation to form an integrated iterative TELL intervention system. This directly resolves the core pain points of Chinese learners in four IELTS Writing Task 2’s scoring sub-criteria. The following discussion interprets the performance effects, user acceptance mechanisms, theoretical innovations, and practical values by engaging in in-depth dialog with existing literature, rather than restating descriptive results.

#### 4.1.1 WETA’s superior intervention effects on IELTS writing performance

The large intervention effect (partial *η*^2^ = 0.715) may be linked to the contextually aligned, low-barrier WETA design integrated with the daily-used Tencent tools, which is consistent with localized EFL intervention studies. Lexical Resources (LR) showed the strongest gain (partial *η*^2^ = 0.526), a result rarely reported in localized TELL interventions for IELTS writing. The result can be attributed to the previously stated WeCWI cycle, WETA’s integrated lexical collocation instruction and topic-based vocabulary resources, and WeCWI’s reading–writing–discussion cycle (Mah et al., 2021) that fosters deep lexical processing as opposed to shallow rote learning. In comparison, traditional IELTS teaching places an excessive focus on the recitation of templates and disjointed vocabulary exercises, which do not result in an improvement in lexical variety and collocational precision (Suo et al., 2025b).

For Task Response (TR, partial *η*^2^ = 0.436), WETA’s prompt-analysis modules and collaborative brainstorming features effectively respond to the longstanding gap in task comprehension and argumentation among Chinese learners (Liang, 2024). This is in line with Arzhadeeva and Kudinova (2020) and Jafary et al. (2023), who affirmed that TR score improvements correlate with the implementation of structured task-analysis and debate-based activities. Contrary to the isolated model-essay interventions (Nguyen and Le, 2022), WETA provides synchronous support via Tencent Meeting and asynchronous feedback through WeChat, which creates an ongoing cognitive activation framework aimed at strengthening learners’ abilities to produce position-focused and well-developed responses.

In Coherence and Cohesion (CC, partial *η*^2^ = 0.412), WETA’s explicit training in logical sequencing and the use of cohesive devices addresses the overuse/misuse of connectors that is common among Chinese EFL learners (Wicaksono et al., 2023). This is in alignment with Sun et al. (2024)‘s finding that thematic progression training has a positive impact on textual coherence, and it further contributes to the field by situating this type of training within a blended synchronous–asynchronous framework. The CG’s minimal improvement in CC corroborates that teacher-centered lectures do not foster organizational competence at the discourse level.

For Grammatical Range and Accuracy (GRA, partial *η*^2^ = 0.389), WETA’s real-time error detection and peer editing technologies are associated with reductions in longstanding grammatical errors with greater efficacy compared to traditional grammar drills. This is in line with Panahi and Mohammaditabar (2015) and Pratama and Munandar (2024), who argued that the efficacy of error correction is higher when it is contextualized, compared to when it is not. The most remarkable aspect of WETA is that its peer + teacher feedback dual mechanism provides greater GRA improvement than feedback from one source (Asadi and Taheri, 2024), which has been a major gap in the IELTS writing instruction.

SLA theory offers a theoretical lens to interpret the potential cognitive mechanisms for the performance gains associated with WeCWI. Reading for Knowledge Construction activates Schmidt’s noticing hypothesis that encourages learners to focus on the IELTS assessment criteria and the features of high-quality writing. The collaborative discussion phase, which is compatible with Long’s interaction hypothesis, allows for the negotiation of meaning and feedback to address logical and linguistic gaps. The iterative cycle of writing and revision addresses Swain’s output hypothesis, leading to the internalization of vocabulary and grammatical structures. Apart from feedback, the cognitive dimension likely underlies WETA’s observed advantage over traditional forms of teaching. As per the quantitative subgroup analysis, WETA’s equity-oriented design is further evidenced by students with low digital literacy, who made significant gains as well.

Considering the large effect size (partial *η*^2^ = 0.715), the observed association between the WETA intervention and IELTS performance is substantial; however, this result must be interpreted with caution due to the quasi-experimental design. While pretest equivalence was established for writing scores, the use of intact classes with non-random assignment means that unmeasured confounding variables—such as differential extracurricular IELTS tutoring, variations in socioeconomic status, prior English-medium instruction, or baseline motivational differences—could not be statistically controlled. Consequently, the large effect size may be partially inflated by these unobserved factors, and the findings should be interpreted as robust associations rather than definitive causal effects. Furthermore, there may be an element of novelty bias. The EG was involved in an extremely structured and technologically mediated ecosystem, which may have temporarily induced hyper-engagement. Extra out-of-class writing time also contributed to this effect, though it was a product of the intervention’s supported environment rather than WETA’s pedagogical design per se. For this reason, the 1.15 band score increase should be interpreted within the context of a highly supported, novelty-driven setting, meaning the score should be viewed as a conservative estimate of the tool’s capability rather than an unquestionable causal proof.

#### 4.1.2 UTAUT-driven acceptance: implications for motivational and affective experiences in EFL writing

While UTAUT traditionally models technology adoption rather than motivational-affective states, its constructs in high-stakes EFL settings may indirectly reflect underlying motivational-affective dynamics. However, it is crucial to reiterate that UTAUT measures user acceptance, not motivational-affective states themselves; any motivational-affective implications are strictly inferential. The qualitative data revealed that low Effort Expectancy was associated with reduced cognitive load and technostress, suggesting a potential, yet unmeasured, alleviation of technology-related friction. More importantly, high Social Influence created a supportive community of practice. This collective engagement is theoretically consistent with a buffering effect against writing anxiety and a catalytic effect on self-efficacy, though these affective states were not directly measured. By bridging the gap between “perceived usefulness” and “empirical effectiveness,” WETA appeared to positively shift learners’ emotional trajectory from exam-driven apprehension to confident engagement. Considering all factors, learners’ evaluations of the use of technology can have an impact on motivations and emotions and impact high-stakes writing practice, including self-efficacy, which is an important motivational component in second language learning (Bandura, 1997). Qualitative findings support that Performance Expectancy, Effort Expectancy, Social Influence, and Facilitating Conditions collectively predict strong Behavioral Intention to use WETA, confirming the UTAUT model’s cross-cultural applicability in non-Western TELL contexts (Venkatesh et al., 2003). This study extends prior UTAUT research in EFL (Nguyen and Chu, 2021; Fitria et al., 2024) by linking perceived acceptance to actual performance gains, resolving the disconnect between “perceived usefulness” and “empirical effectiveness” in existing edtech acceptance studies. These findings align with recent research on writing anxiety in Asia-Pacific EFL contexts, where high-stakes test pressure has been widely documented as a key barrier to sustained writing engagement (Liang, 2024; Suo et al., 2025b). The low-anxiety, collaborative learning environment fostered by WETA thus addresses a critical affective pain point for Chinese IELTS learners, which has long been overlooked in traditional teacher-centered instruction.

Performance Expectancy of students emerged as the most dominant driver of user acceptance, as reflected in the highest coding node count (*n* = 46), with participants consistently linking WETA use to measurable improvements in IELTS writing performance. Grounded in Social Cognitive Theory (Bandura, 1997), such perceived performance gains implicitly translate into enhanced academic self-efficacy; as learners perceive the tool as instrumentally effective (Venkatesh et al., 2003), their belief in their own capability to succeed in high-stakes writing is correspondingly reinforced. Effort Expectancy was the second strongest facilitator (*n* = 17), as WETA’s integration with WeChat and Tencent Meeting—platforms used daily by over 90% of Chinese university students(Qin and Yu, 2024; Wang and Luo, 2022)—drastically lowered the learning threshold and adoption barriers. Learners mastered core functions in 15–20 min, which supports Yan (2019) and Luo et al. (2023)‘s conclusion that familiarity with local digital tools drastically lowers adoption barriers. This reduced cognitive load and technostress are theoretically consistent with alleviating foreign language writing anxiety (Horwitz et al., 1986). When technological friction is minimized (Venkatesh et al., 2012), learners can allocate cognitive resources to the writing task itself rather than navigating the platform, thereby implicitly lowering the affective filter associated with EFL writing. This finding challenges the dominance of Western edtech platforms in TELL research, proving that locally embedded tools achieve higher usability and sustainability (Alenazi, 2023; Masoud, 2024).

In the case of the collectivist Chinese learning context, Social Influence was a primary driver of student acceptance, as peer collaboration and teacher scaffolding created a motivating environment. Viewed through the lens of Self-Determination Theory (Deci and Ryan, 2000), this social support fulfills learners’ psychological needs for relatedness and competence. Consequently, while UTAUT measures Social Influence as an external driver of acceptance, it implicitly catalyzes internalized foreign language learning motivation, shifting learners from extrinsic compliance to intrinsic engagement. Importantly, while the instructor’s separate qualitative reflection confirmed the implementation feasibility of WETA, it was the students’ own reports of peer and teacher support that constituted the evidence for acceptance (Asadi and Taheri, 2024; Al-Yafaei and Mudhsh, 2023; Mohammad-Salehi et al., 2021), thereby avoiding conflation of instructor and student perspectives. WETA’s peer review and collaborative discussion features shifted the focus from individual learning to collective learning, which is a neglected design principle in most Western TELL tools (Alhassan et al., 2021). This peer-driven social reinforcement is theoretically consistent with satisfying learners’ self-determination needs, which catalyzes intrinsic motivation in EFL settings (Hsu, 2023), and aligns with findings that collaborative writing environments significantly enhance writing self-efficacy (Truong, 2022).

Facilitating conditions such as stable hardware, 24/7 tech support, and support across different devices removed practical problems to use tools (Kim and Lee, 2022; Ma et al., 2022) and is also the practical support these authors refer to in their research. Crucially, according to the Control-Value Theory of Achievement Emotions (Pekrun, 2006), such enabling conditions implicitly shape learners’ achievement emotions. By ensuring stable, equitable access (e.g., offline caching) and removing technical barriers, WETA enhances learners’ perceived control over the learning environment. This heightened control is a primary antecedent for generating positive achievement emotions (e.g., hope, enjoyment) and mitigating negative ones (e.g., hopelessness, anxiety). WETA alleviated concerns of the digital divide with its offline caching and low-data-consuming design, which supports UNESCO (2023) equity focus on the global edtech agenda.

Beyond the perceptual data derived from the UTAUT interviews, the quantitative engagement metrics (Figure 2) provide crucial behavioral evidence supporting the implied motivational and affective benefits. The EG demonstrated significantly high frequencies in reading posts, writing submissions, discussion comments, and peer feedback entries. In the context of TELL research, such high-frequency interactive behaviors serve as robust, objective indicators of behavioral engagement (Fan et al., 2023; Fredricks et al., 2004). When triangulated with the qualitative finding that Social Influence—manifested through peer collaboration and feedback—was a primary driver of acceptance, these activity post counts act as observable manifestations of enhanced behavioral engagement and social learning motivation (Muluk et al., 2022), rather than direct measures of affective states. While the CG’s lack of digital posts reflects the absence of interactive channels in traditional instruction rather than a lack of motivation, the WETA group’s sustained out-of-class interactions indicate that the tool did not merely change how students learned, but also increased how much they were willing to actively participate in the learning community. This behavioral triangulation provides robust corroborating evidence for the UTAUT-driven implication that WETA contributes to cultivating a motivating, potentially low-anxiety collaborative environment.

While this study did not harness standardized psychological inventories to directly measure the affective, the triangulation of quantitative behavioral engagement data (high-frequency interaction post counts), qualitative thematic evidence (UTAUT-driven interview coding on reduced apprehension), and objective performance gains (ANCOVA results) offers robust, multi-dimensional corroboration for the implied motivational-affective benefits. To be specific, The EG’s sustained interactions (Figure 2) serve as objective indicators of active participation and reduced behavioral avoidance. While reduced avoidance is theoretically associated with lower writing anxiety, activity metrics cannot directly measure affective states. Hence, while the motivational-affective improvements remain ‘implied’ and hypothesis-generating in the absence of direct psychometric scaling, the convergent evidence of high user acceptance (UTAUT) and high behavioral engagement (post counts) provides a rationale for future psychometric testing of WETA’s potential impact on learner affect.

#### 4.1.3 Theoretical contributions: WeCWI-ADDIE-UTAUT integrated framework

The research makes three distinct contributions to TELL and IELTS pedagogy.

First, it demonstrates the feasibility of the WeCWI-ADDIE-UTAUT integrated framework conceptualized by the researchers for TELL tool development in non-Western contexts, expanding Mah et al. (2021) WeCWI model for high-stakes EFL writing and adjusting the western-centric UTAUT model to accommodate Chinese collectivist learning contexts. Most other TELL frameworks focus either on design (Branch and Merrill, 2012) or user acceptance (Venkatesh et al., 2012) in isolation. This study connects design, implementation, effectiveness, and user acceptance. While this study demonstrates the feasibility of integrating the WeCWI, ADDIE, and UTAUT frameworks, it is important to clarify that the current study applies these frameworks sequentially rather than testing them as a unified structural model. Future research employing structural equation modeling (SEM) is needed to validate the latent constructs and path relationships within this integrated framework.

Second, it attempts to explain the “black box” of technology-enabled writing improvement (Moore et al., 2016) by relating specific cognitive processes involved in TR/CC/LR/GRA to WETA features (screen-sharing, peer review, vocabulary banks and so forth). This answered the “how” of TELL rather than the “whether it works” of mechanism-focused TELL research (Hyland, 2019).

Third, it develops a theory of equitable TELL, demonstrating that low-cost, locally available tools can produce sizable results without the expensive infrastructure often thought necessary, debunking the misconception that effective edtech relies on proprietary Western technologies (Alem, 2019; Zandi and Krish, 2017). The subgroup findings from this study refine this theory by showing that for TELL design to be equitable, it must prioritize alignment to learners’ digital habits (e.g., daily use of WeChat/Tencent Meeting) to remove barriers to adoption, rather than expecting learners to use new, unfamiliar platforms. This expands WeCWI-based TELL research to include marginalized learners who possess lower digital literacy, a demographic that previous edtech intervention research has disregarded.

Notably, this research provides empirical evidence supporting the synergistic mechanism of the WeCWI-ADDIE-UTAUT framework, which was only theoretically described in earlier studies, offering the first empirical evidence supporting its feasibility in high-stakes EFL writing contexts.

#### 4.1.4 Practical and educational equity implications

WETA offers a low-cost model where direct financial barriers for students are minimized, allowing IELTS writing instruction to be implemented in Chinese universities and other matching EFL teaching contexts. WETA, unlike the AI writing tools (Chen et al., 2025), which demand a high level of digital literacy, is designed for learners of lower digital literacy, therefore narrowing the outcomes gap in regard to proficiency (Cheng et al., 2025; Huang et al., 2023). For teaching professionals, WETA transforms the role from knowledge supplier to knowledge facilitator, which is in line with Nguyen and Stracke (2023) and their blended EFL teaching model.

While WETA avoids the high licensing fees of proprietary Western platforms, it is important to clarify that it is a low-cost rather than zero-cost solution. Its implementation requires acknowledging real institutional investments, including instructor training time, Tencent Meeting subscriptions, curriculum development labor, and technical support staffing. Nevertheless, the low-cost, local nature of WETA removed barriers for students with few resources, which aligns with the equity outcomes described in the report. For educational equity, the subgroup outcomes from this research provide evidence that WETA narrows the digital gap for EFL writing instruction, as even learners with low initial digital literacy achieved writing performance gains comparable to their more digitally literate peers without any additional technical training, just 15 min of initial guidance was all that was needed.

This research directly addresses the Global South’s longstanding edtech conundrum: the commercially available western tools are expensive, inconsistent with the local context, and demand a high level of digital literacy, whereas the local solutions have historically lacked a robust pedagogical framework and empirical evidence (Alenazi, 2023; Alem, 2019). This research offers a framework that is potentially adaptable for the creation of context-relevant TELL tools and illustrates potential alignment with the goals of UN SDG 4 (Quality Education) as proposed by UNESCO (2023) and Alenazi (2023).

### 4.2 Conclusion

This study indicates that WETA—an integrated TELL tool combining WeCWI pedagogy, WeChat, Tencent Meeting, and UTAUT-driven user design—is associated with significant improvements in Chinese EFL undergraduates’ IELTS Writing Task 2 performance across its four IELTS scoring sub-criteria, with high user acceptance and strong behavioral intention for sustained use. The quasi-experimental results (large effect sizes, ANCOVA *p* < 0.001) and qualitative UTAUT findings collectively suggest that theory-driven, locally embedded blended tools are associated with better outcomes compared to traditional teacher-centered instruction for high-stakes EFL writing.

The significance of this study transcends mere pedagogical efficiency; it highlights the necessity of considering motivational-affective dimensions in TELL design. Future research should continue to explore the longitudinal trajectory of learner’s academic resilience and writing engagement within such technology-mediated, emotion-aware instructional ecosystems.

The core contributions are:

1. Providing empirical evidence for the feasibility of a holistic TELL intervention that links pedagogical theory, local digital infrastructure, and user acceptance;
2. Refining UTAUT for collectivist EFL contexts by highlighting effort expectancy and social influence as key drivers;
3. Highlighting how theory-driven TELL interventions may imply potential reductions in writing anxiety and be associated with increased self-efficacy, serving as hypothesis-generating findings that require direct psychometric validation;
4. Providing an equitable, low-cost edtech model for the Global South to reduce digital and educational gaps;
5. WETA addresses long-standing pain points in IELTS writing instruction and sets a new standard in context-suitable, theory-driven, equity-focused TELL tool development.

### 4.3 Limitations

This study has recognized a number of theoretical and methodological constraints that limit how its results can be interpreted.

Theoretically, the first constraint is the WeCWI-ADDIE-UTAUT framework’s boundary conditions, which remain indeterminate. The model has only been tested for feasibility in the context of Chinese EFL undergraduates’ IELTS Academic Writing Task 2, so its transferability to other second language (L2) writing sub-domains (TOEFL writing and graduate academic writing) or to other learner demographics has yet to be verified. The second factor is that this study is concerned only with the four core constructs of UTAUT and has not considered the moderating constructs as of yet (e.g., baseline second language (L2) proficiency and digital literacy) and this has put certain limitations on how far the localized refinement of the UTAUT framework can be taken.

Methodologically, the first factor is that although baseline equivalence was established via pretests (*p* > 0.05), this quasi-experimental design using intact classes with non-random assignment cannot fully eliminate selection bias or unmeasured confounding variables common in Chinese high-stakes test preparation settings, such as differential extracurricular IELTS tutoring, variations in socioeconomic status, prior English-medium instruction, or baseline motivational differences. While ANCOVA controlled for pretest scores, it could not account for hidden external investments in test preparation. Thus, attributing the observed effect size of 0.715 solely to the WETA tool must be cautious, as intact class assignment cannot statistically isolate its pure causal effect from real-world confounding factors, which diminishes the extent to which causality can be ascertained when compared to a fully randomized controlled trial (Buitrago and Díaz, 2017; Muluk et al., 2022). Furthermore, unmeasured extracurricular IELTS tutoring outside the classroom could not be ruled out as a hidden confounding factor inflating the effect size. Additionally, while formal contact time was strictly controlled at 2 h per week for both groups, the WETA environment fostered voluntary out-of-class engagement (e.g., additional peer feedback on WeChat as shown in Figure 2). This unmeasured variance in self-regulated learning time is a potential, though motivationally justified, confounding factor, as enhanced voluntary engagement is an intended outcome of the intervention’s motivational design rather than an instructional dosage requirement. The second factor is that the findings of this research can only be applicable to the 60 undergraduates from a University and no other students from different geographical, institutional, or age backgrounds can be included. The third factor is the 8-week intervention which only looks at short-term performance improvement and does not have a long-term measurement to determine whether the learning outcomes or the sustained use of the tool can be maintained to disentangle the true pedagogical effect of WETA from novelty and other confounding effects. The fourth methodological factor is that the motivational-affective benefits discussed herein remain implied and hypothesis-generating due to the absence of direct psychometric assessment using standardized validated pre/post instruments (e.g., SLWAI for writing anxiety; Academic Self-Efficacy Scale). As UTAUT constructs measure technology acceptance and activity post counts measure behavioral engagement, neither can serve as valid proxy indicators for motivational-affective states. Hence, any claims regarding anxiety or self-efficacy are inferential and require direct validation. Additionally, the fifth methodological constraint pertains to the utilization of an identical writing prompt for both the pretest and posttest. While retaining a consistent topic was a deliberate methodological choice to prevent the conflation of topic effects with the intervention effect, this repetition potentially introduced a practice or familiarity effect. Participants who had previously engaged with the prompt during the pretest might have approached the posttest with enhanced topical fluency. Consequently, a proportion of the observed score gains may be attributable to this familiarity advantage rather than the intervention itself. Subsequent research might consider counterbalancing alternate prompts of equivalent difficulty across testing occasions to disentangle practice effects from the true pedagogical intervention.

### 4.4 Future research

Considering the findings and limitations of this study, we outline four specific, theory-based suggestions for subsequent research. First, the WeCWI-ADDIE-UTAUT framework should be subjected to replication studies in various L2 writing situations, learner demographics, and non-Western EFL contexts, in order to specify its boundary limitations and improve its usability across different contexts. Second, subsequent research should aim to establish stronger causal claims regarding the intervention’s impact outcomes by employing fully randomized controlled trial (RCT) designs with larger sample sizes across multiple sites. Specifically, to disentangle the observed associations of WETA from novelty bias from novelty bias and real-world confounding factors, future studies must track extracurricular IELTS tutoring as a covariate, thereby assessing a more conservative and generalizable effectiveness of WETA (Peng and Raman, 2026). Third, researchers can utilize the extended localized UTAUT model to explain condition L2 proficiency and digital literacy in collectivist EFL contexts as moderating variables and to employ structural equation modeling to describe the different layers of technology use. Finally, for further qualitative research, perspectives of different stakeholders should be included, such as teachers with various backgrounds, language assessment professionals, and school administrators, to offer a broader view of the strengths and weaknesses of the WETA model in various practical teaching contexts. Furthermore, to move beyond implied motivational-affective benefits, subsequent research must integrate standardized psychological inventories like the Second Language Writing Anxiety Inventory to empirically test the hypothesized reduction in writing anxiety and the potential enhancement of self-efficacy suggested by the current behavioral and qualitative data.

## Data Availability

Anonymized raw data that support the findings of this study are available from the corresponding author upon reasonable request, subject to institutional data protection restrictions.

## Appendix

### Appendix A: IELTS Writing Task 2 prompts used in pretest and posttest

The pre-test prompt:

Some people think that watching television is helpful for children. To what extent do you agree or disagree with it?

*You have 40 min to write this essay with your examples, experience, etc. and the word limit is around 250 words*.

The post-test prompt:

Some people think that watching television is helpful for children. To what extent do you agree or disagree with it now?

*You have 40 min to write this essay with your examples, experience, etc. and the word limit is around 250 words*.
